# Investigation of the Cognitive Attitudes and Behaviors of Medical Post-graduates in Clinical Practice During the COVID-19 Pandemic in China

**DOI:** 10.3389/fpubh.2021.755163

**Published:** 2021-11-03

**Authors:** Lei Feng, Zefeng Xie, Junhui Shen

**Affiliations:** Eye Center, Second Affiliated Hospital, School of Medicine, Zhejiang University, Hangzhou, China

**Keywords:** COVID-19 pandemic, medical post-graduates, clinical practice, attitudes and behaviors, investigation

## Abstract

The cognitive attitudes and behaviors of medical post-graduates may be influenced by the coronavirus disease 2019 (COVID-19) pandemic. A cross-sectional study was used from a questionnaire survey in hospitals affiliated with the Zhejiang University School of Medicine. Questionnaire was distributed online including demographic information, cognitive attitudes, and personal protective behaviors. Moreover, personal protective behaviors such as wearing protective equipment were compared between different academic major and gender, respectively. A total of 176 valid questionnaires were obtained. Of the medical post-graduates in this study, (1) 89.67% believed that the COVID-19 pandemic had an impact on their clinical internships, and 40.34% expressed concerns about their infection on inadequate personal protection; (2) 91.48% took personal protection in hospital and 86.36% enhanced personal hygiene; (3) There were no statistically differences in the personal protection by academic major and gender (*p* > 0.05). This study suggests that the COVID-19 pandemic had an impact on the medical post-graduates' clinical practice, and affected their cognitive attitudes and behaviors. As such, universities and hospitals should increase pandemic prevention training and investment, provide more psychological counseling to their medical post-graduates to reduce their psychological burden, and take measures to reduce the influence of the COVID-19 pandemic on their medical post-graduates' clinical practice.

## Introduction

Since the World Health Organization announced in January 2020 that the coronavirus disease 2019 (COVID-19) pandemic is a public health emergency of international concern (1), the Chinese medical staff have demonstrated outstanding skill and dedication in striving to save lives across the country. COVID-19 has caused unprecedented damage to the medical education system worldwide (2). It is difficult to continue teaching as usual, thus affecting the lecture and patient-based clinical practice (3). The COVID-19 pandemic puts people at risk, which poses a major challenge to medical education, because teachers must teach safely while at the same time ensuring the integrity and continuity of the medical education system. Due to the focus on patients with COVID-19, these challenges have resulted in limited patient care, which limits the opportunities for medical students to teach at the bedside (4). Clinical practice through rotation has been suspended for a long time (5). Other challenges include concerns that medical students may contract the virus during their clinical practice and need to wear personal protection (6). Passing on their skill and dedication to the future medical practitioners is the most important task of medical education. In consequence, COVID-19 may cause great impact on the medical students' attitude and behaviors during the clinical practice (7, 8).

As of this writing, as the COVID-19 pandemic is under control in China, clinical medical post-graduates have returned to the hospital to carry out their internships. However, due to the normalization of the pandemic prevention, the medical post-graduates who are preparing for medical practice are not only striving to learn what they should but are also enduring the long-term challenges posed by the COVID-19 pandemic. There is a growing literature on how medical educators and students adapted to the COVID-19 pandemic (9–13). However, the studies from China are limited. In this study, the cognitive attitudes and behaviors of medical post-graduates conducting internship during the COVID-19 pandemic in China were investigated through a questionnaire survey. Our research questions are:

1. What are the clinical medical post-graduates' cognitive attitudes toward the COVID-19 pandemic?
2. What are the personal protective measures taken by the clinical medical post-graduates in response to the COVID-19 pandemic?
3. What are the difference of prevention and control measures taken by clinical medical post-graduates with different majors?
4. What are the difference of prevention and control measures taken by clinical medical post-graduates with different genders?

## Methods

### Participants

The subjects of this study were clinical medical post-graduates of the Zhejiang University School of Medicine. We conducted a cross-sectional survey from March to April, 2021.

### Data Collection From the Questionnaire Survey

The questionnaires had 3 parts. The first part asked about the basic demographic information of the respondents (such as gender, age, grade, academic major, and health status). The second part was the questionnaire addressed their experience with cognitive attitudes during the COVID-19 pandemic, including the impact of COVID-19 on clinical practice, reasons for anxiety and fear during clinical practice, worry in clinical practice during the COVID-19 pandemic, the most desirable COVID-19 prevention and training mode, most desired COVID-19-related training content, vaccination, and most gratified thing about the current pandemic prevention and control. The third part was the investigation of personal protective measures of the clinical medical post-graduates. They were asked about which kinds of personal protective measures to take, such as wearing protective articles in the hospital, strengthening one's personal hygiene, reducing access to patients, and exercising more to improve one's immunity.

We developed the questionnaire in Chinese. We tested its internal consistency in a pilot study comprising 50 students. All the questionnaires had high level of internal consistency among our study population, with a Cronbach's alpha over 0.9 (0.91, 0.94, 0.96, respectively). An electronic survey questionnaire was formulated and randomly sent out through the Questionnaire Star survey program (https://www.wjx.cn/) on WeChat. A total of 195 questionnaires were sent out. Nineteen of the retrieved questionnaires were invalid and were thus excluded from the study, and 176 were valid. The effective survey questionnaire retrieval rate was thus 90.26%.

### Statistical Analysis

The data from the retrieved electronic survey questionnaires were gathered by Questionnaire Star. We described categorical variables as frequencies and percentages. The data were analyzed using chi-square tests (χ^2^) to compare the differences between groups and *p*-values were two-sided and considered significant at *p* < 0.05. All data were analyzed by SPSS version 22.0 software.

### Ethical Approval

Ethical approval was obtained from the ethics committee of the Second Affiliated Hospital, School of Medicine, Zhejiang University in China. All participants provided written informed consent prior to participating in the study, without identifiable data.

## Results

### Demographic Characteristics

A total of 176 medical post-graduates completed the survey, and most of them (57.95%) were female. Table 1 shows the distribution of the study participants. Most of those who completed the survey were post-graduate students of ophthalmology, otorhinolaryngology, and stomatology (43.75%). The current health status of the students was basically good, and the proportion of students with a poor health status was only 1.7% based on self-assessment.

**Table 1:** Demographic characteristics of the study subjects.

**Essential information**	**Classification**	**No. of**	**Percentage**
		**people**	**(%)**
Gender	Male	74	42.05
	Female	102	57.95
Grade level	First year, master's program	45	25.57
	Second year, master's program	47	26.70
	Third year, master's program	36	20.45
	First year, doctoral program	15	8.52
	Second year, doctoral program	14	7.95
	Third year, doctoral program	11	6.25
	Fourth year, doctoral program	8	4.55
Major	Ophthalmology, otorhinolaryngology, stomatology	77	43.75
	Internal medicine	63	35.80
	Surgery	20	11.36
	Laboratory science, medical imaging	13	7.39
	Nursing	3	1.70
Home location	Town	101	57.39
	Countryside	75	42.61
Current health status	Very good	62	35.23
	Good	76	43.18
	General	35	19.89
	Poor	3	1.70

### Cognitive Attitudes of Medical Post-graduates in Clinical Practice During the COVID-19 Pandemic

Of the medical post-graduates who participated in this study, 89.77% believed that the COVID-19 pandemic had affected their clinical practice in different ways (Table 2). Among these was the fact that they developed nervousness and fear due to their knowledge that the virus causing COVID-19 could be transmitted in various ways and was highly infectious. Their biggest worries in relation to their clinical practice were self-infection, cross-infection, and large-scale infection due to inadequate personal protection, the presence of too many patients in the hospital, and the patients' concealment of their illness. For pandemic prevention and training mode, most of the students hope to obtain is the unified arrangement of the medical school and the hospital. Most of the students (63.64%) thought that the COVID-19 vaccines were effective, and 66.5% thought that being vaccinated is the best way to gradually return to original clinical work.

**Table 2:** Results of the questionnaire survey on the clinical medical post-graduates' cognitive attitudes toward the COVID-19 pandemic.

**Questionnaire content**	**No. of people**	**Percentage (%)**
**Impact of COVID-19 on your clinical practice:**		
Slightly affected	81	46.02
Moderately affected	44	25.00
Greatly affected	27	15.34
Not affected	18	10.23
Very much affected	6	3.41
**Reasons for anxiety and fear during clinical practice:**		
COVID-19 can be transmitted in many ways.	51	28.98
No fear	40	22.72
The virus is too infectious.	39	22.16
Lack of effective treatment for COVID-19	32	18.18
There are more severe pneumonia patients and there is a higher mortality rate during the pandemic.	14	7.95
The family burden is increased by COVID-19 infection.	3	1.70
**What makes you worry in clinical practice during the COVID-19 pandemic (only can choose one answer):**		
Self-infection may occur when one is not wearing personal protective gear.	71	40.34
Cross-infection can easily occur as there are too many floating personnel in the hospital.	52	29.55
Large-scale infection may be caused by the patients' concealment of their condition.	46	26.13
The COVID-19 pandemic complicates the original clinical work.	7	3.98
**The most desirable COVID-19 prevention and control training mode is:**		
Unified arrangement of study in the school and the hospital	153	86.93
Autonomous online learning	23	13.07
**Most desired COVID-19-related training content:**		
Related operation process	77	43.75
Personal protection	66	37.50
Disinfection and isolation	33	18.75
**Do you think vaccination has a preventive effect?**		
Yes	112	63.64
Usually	62	35.23
No	2	1.14
**What makes you gratified about the current pandemic prevention and control (only can choose one answer)?**		
Life is gradually getting back on track.	117	66.48
The public awareness of the need for personal protection has been strengthened.	19	10.80
The number of new cases has decreased.	15	8.52
The government has taken effective measures to control the spread of the virus causing COVID-19.	11	6.25
There is progress in the new-drug research and development.	10	5.68
The number of cured cases has increased.	3	1.70
Other	1	0.57

### Personal Protective Measures and Behaviors of Clinical Medical Post-graduates During the COVID-19 Pandemic

Of the medical post-graduates who participated in this study, 161 (91.48%) took protective measures against COVID-19 infection in the hospital during their internship (Table 3). One hundred fifty-two medical post-graduates (86.36%) chose to strengthen their personal hygiene, and 61 (34.66%) chose to reduce their contact with the patients. The number of medical post-graduates who exercised more to improve their immunity was basically the same as the number of those who did not take such measure. Seven medical post-graduates (3.98%) did not take any measure to prevent themselves from acquiring COVID-19.

**Table 3:** Survey questionnaire items on the personal protective measures taken by the clinical medical post-graduates in this study during the COVID-19 pandemic.

**Personal protective measures**	**No. of**	**Percentage**
	**executors**	**(%)**
Wearing protective articles in the hospital	161	91.48
Strengthening one's personal hygiene	152	86.36
Reducing access to patients	61	34.66
Exercising more to improve one's immunity	82	46.59
None	7	3.98

### Prevention and Measures Taken by Clinical Medical Post-graduates With Different Majors and Genders

Table 4 shows that there was no statistically significant difference in taking various prevention and measures among the medical post-graduates in this study by academic major (*p* > 0.05). Table 5 shows that the proportion of male and female medical post-graduates who wear protective items in hospitals exceeds 90%. Interestingly, in terms of strengthening personal hygiene, the percentage of males who did so (83.78%) was slightly lower than the percentage of females who did the same (88.24%). There was no significant difference in the prevention and measures taken by gender (*p* > 0.05).

**Table 4:** Comparison of the prevention and control measures taken by academic major.

**Protective measures taken**	**Academic major**					**χ^2^**	***p***
	**Ophthalmology, otorhinolaryngology, stomatology (*n*/%)**	**Internal medicine (*n*/%)**	**Surgery (*n*/%)**	**Nursing (*n*/%)**	**Medical imaging (*n*/%)**		
Wearing protective articles in the hospital	74/96.10	54/85.71	18/90	3/100	12/92.31	5.145	0.273
Strengthening personal hygiene	72/93.51	52/82.54	14/70	3/100	11/84.62	9.173	0.057
Reducing access to patients	23/29.87	24/38.10	7/35	0	7/53.85	4.814	0.307
Exercising more to improve immunity	35/45.45	25/39.68	10/50	1/33.3	11/84.62	9.107	0.058
None	1/1.30	6/9.52	0	0	0	–	–

**Table 5:** Comparison of differences in the prevention and control measures taken by gender.

**Protective measures taken**	**Gender**		**Total (*n*/%)**	**χ^2^**	***p***
	**Male (*n*/%)**	**Female (*n*/%)**			
Wearing protective articles in the hospital	67/90.54	94/92.16	161/91.48	0.144	0.705
Strengthening personal hygiene	62/83.78	90/88.24	152/86.36	0.722	0.396
Reducing access to patients	28/37.84	33/32.35	61/34.66	0.570	0.450
Exercising more to improve immunity	37/50.00	45/44.12	82/46.59	0.596	0.440
None	4/5.41	3/2.94	7/3.98	0.682	0.409

## Discussion

The COVID-19 pandemic has swept the world, and the pandemic prevention and control work has become a norm (14). As they serve as the backup force of clinicians, medical post-graduates consider clinical practice the main content of their study. However, due to the large number of patients in hospitals and the high work intensity, their occupational exposure risk from the doctor–patient ratio imbalance is much higher than that of students in other academic majors (15). The changeable clinical situation will create tremendous pressure on clinical medical students who are still in the growth stage, and this is likely to affect their clinical study and practice (16). During the COVID-19 pandemic, especially in the stage of normalization of the pandemic prevention and control work, medical post-graduates in clinical practice are faced with clinical risks and thus belong to the high-risk groups. This study focused on the cognitive attitudes and prevention and control behaviors of clinical medical students during the COVID-19 pandemic. According to the survey, the pandemic has had an impact on the medical post-graduates' clinical practice, but the students have a relatively positive attitude toward the pandemic situation. They are also doing a good job of protecting themselves in their clinical practice. For hospitals and schools, they should strengthen their personal protection training, encourage active participation in vaccination, and strengthen the teaching of infectious-disease prevention and control. At the same time, they should actively cultivate the professional quality and identity of medical post-graduates.

The survey showed that 89.77% of the medical post-graduates thought that the COVID-19 pandemic has had an impact on them. Among these, 15.34% thought that the pandemic has had a great influence and 3.41% thought that the pandemic has had a major influence on their clinical practice. The reasons for these can be roughly divided into two points. First, in early 2020, when the domestic pandemic situation was relatively severe, almost all the medical students in clinical practice were required to stop their practice for their own protection (17). They resumed their clinical practice after several months, and no measures were put in place to make up for their lack of practice time. Second, some clinical frontline doctors were transferred to pandemic prevention and control teams, leading to a decrease in the number of clinical teachers (18). As each of the remaining clinical teachers was then put in charge of more students to make up for the shortage of clinical teachers, the teaching effect was bound to be affected (19). With the emergence of COVID-19 vaccines and vaccination, 66.5% of the medical students were glad to see that life was getting back to the old normal, which showed that most of the students were eager to return to normal life after experiencing the pandemic. Schools need to encourage their students to take part in the pandemic prevention and control training and work and to strive to cut off the COVID-19 transmission routes and to get back to the old normal as soon as possible.

A previous study found that the COVID-19 pandemic has had a negative psychological impact on post-graduate trainees (20). Among Chinese college students, 0.9% suffer from severe anxiety, and 2.7% have moderate anxiety symptoms during the outbreak of COVID-19 pandemic (21). We found that in spite of the pandemic, the post-graduates in all the academic departments and grades in our University are basically in good health. A high 78.41% of the students said that they were in good health, which shows that the pandemic prevention and control work in our University has yielded relatively satisfactory outcomes. COVID-19 has had little influence on the health status of the University's medical clinical post-graduates. This study also investigated the infection prevention measures that could be taken by clinical post-graduates. It was found that medical students are more conscious of their personal protection in the face of the pandemic. Only 3.98% of the students said that they had not taken any preventive measure during the pandemic, and the remaining students said that they had taken different protective measures against COVID-19 infection. Moreover, there were no significant differences in the prevention and control measures taken by the medical post-graduates by gender and academic major. The results showed that 91.48% of the medical post-graduates chose to strengthen their personal protection against COVID-19 and 86.34% chose to strengthen their personal hygiene. Previous studies have demonstrated that the hands are an important medium for virus transmission, and hand hygiene is thus the simplest and most cost-effective measure for reducing the occurrence of hospital infection (22). Hand hygiene is also listed in the guidelines issued by China to protect the public against COVID-19 as one of the important measures that can be taken to prevent COVID-19 infection (23). However, only 46.59% of the students chose to exercise more to improve their immunity and to reduce their risk of infection. Zhu et al. found that it is safe to exercise during the COVID-19 outbreak (24). Proper physical exercise is also a scientific protective measure to strengthen one's resistance. Our study results also showed that most of the students had a positive attitude toward the COVID-19 vaccine, and only 1.14% of them thought that the vaccine had no effect on the prevention of COVID-19 infection. The existing literature also confirmed that the vaccine used in China has good immunogenicity (25). In the follow-up pandemic prevention and control work, accelerating the maturity and popularization of the existing vaccines and increasing the COVID-19 vaccination will play a relatively positive role in combatting the COVID-19 pandemic. Thus, medical schools and hospitals can also strengthen their training programs on and investments in personal protection and personal hygiene for the medical post-graduates, encourage the medical post-graduates to actively engage in physical exercise, provide safe exercise spaces for them, and actively promote COVID-19 vaccination among them to reduce the impact of the pandemic on their clinical practice and to better protect them against COVID-19 infection.

COVID-19 is a new infectious respiratory disease. Due to its multiple transmission routes and strong contagion effect (25), the biggest worry of the medical post-graduates in this study was their COVID-19 infection due to their inadequate personal protection and the possibility of cross-infection in the hospital. The survey also found that 86.93% of the students hope that unified arrangement of pandemic prevention and control training mode can be achieved. There have been three major disease outbreaks in the 21st century: China's severe acute respiratory syndrome outbreak (26) in 2003, the Middle East respiratory syndrome (MERS) outbreak in 2012 (27), and the West African Ebola epidemic in 2014 (28). Thus, it is possible that every medical post-graduate student in clinical practice will face a new infectious-disease outbreak. Therefore, knowledge regarding pandemic prevention and control and the related training are necessary in hospitals and medical schools. It is also one of the most recognized forms of training for medical post-graduates. In recent years, with the advances in the field of medicine, the spectrum of infectious diseases has changed significantly; for instance, the incidence rates of plague, cholera, and encephalitis B have dropped significantly (29). The COVID-19 pandemic has shown the need to teach infectious-disease outbreak control as new infectious diseases may emerge at any time. Every clinical worker should thus be alert to the emergence of new infectious diseases. For the teaching of infectious-disease treatment and control for medical post-graduates, what is most important is to update the information in real time and to add the concept, types, characteristics, causes, and influencing factors of new infectious diseases to the medical syllabus on infectious diseases. Second, it is necessary to increase the exercises in coping with the outbreak of infectious diseases in the teaching of the said matter so that the students will be able to understand all the aspects of the prevention and control of infectious diseases through actual participation. Combined with the existing resident standardized training, hospitals can also increase the training contents related to infectious diseases and pandemic response.

In early 2020, the COVID-19 pandemic raged around the world (30, 31). Tens of thousands of Chinese medical staff rushed to Wuhan, China, to help treat the patients there, bravely facing the risk of infection and demonstrating the resolve to “respect life, save the dead, and heal the wounded” (32). As the reserve army of clinicians, medical students have the mission not only to safeguard human health but also to inherit the spirit of medical practice. Therefore, besides medical skills, moral education should be placed at the core of medical education (32). Before medical students start their clinical practice, teachers should make them feel the suffering of patients at close range, experience the race against death, and actively cultivate their professionalism and professional identity. The results of this survey showed that most of the medical post-graduates in this study were not willing to reduce their risk of contracting COVID-19 by reducing their contact with patients. The medical education curriculum can regard pandemics as a teaching material and can mandate the discussion of these in class, along with medical skills and medical ethics education (33).

## Conclusions

In conclusion, our study found that the COVID-19 pandemic had an impact on the medical post-graduates' clinical practice, and affected their cognitive attitudes and behaviors. There were no statistically differences in the personal protection by academic major and gender. Medical schools and hospitals should therefore increase their targeted measures against the medical post-graduates' COVID-19 infection, offer psychological counseling to them to lighten their psychological burden, and strive to reduce the impact of outbreaks on their clinical practice.

## Data Availability Statement

The raw data supporting the conclusions of this article will be made available by the authors, without undue reservation.

## Ethics Statement

The studies involving human participants were reviewed and approved by the Ethics Committee of the Second Affiliated Hospital, School of Medicine, Zhejiang University, and conducted in accordance with the Declaration of Helsinki principles. Informed consent from all participants was included at the start of the questionnaire, and all participants voluntarily participated or withdrew from the questionnaire. Written informed consent to participate in this study was provided by the participants' legal guardian/next of kin.

## Author Contributions

LF and JS: conceptualization and formal analysis, funding acquisition, and writing—review and editing. ZX: data curation. LF, ZX, and JS: methodology. LF and ZX: writing—original draft. All authors contributed to the article and approved the submitted version.

## Funding

This study was supported by the National Natural Science Foundation of China (Nos. 81870648, 82070949, and 82000886) and Natural Science Foundation of Zhejiang Province (No. LQ20H120011).

## Conflict of Interest

The authors declare that the research was conducted in the absence of any commercial or financial relationships that could be construed as a potential conflict of interest.

## Publisher's Note

All claims expressed in this article are solely those of the authors and do not necessarily represent those of their affiliated organizations, or those of the publisher, the editors and the reviewers. Any product that may be evaluated in this article, or claim that may be made by its manufacturer, is not guaranteed or endorsed by the publisher.
